# The enigma of squamous cell carcinoma of the colon: a case report and review

**DOI:** 10.3389/fonc.2026.1741167

**Published:** 2026-02-11

**Authors:** Eesha Oza, Yashvi Rateshwar, Areeba Nayyer, Steven Brower

**Affiliations:** 1 Albert Dorman Honors College, New Jersey Institute of Technology, Newark, NJ, United States; 2 Bergen County Academies, Hackensack, NJ, United States; 3 Department of Medicine, Englewood Health, Englewood, NJ, United States; 4 Department of Surgery, Englewood Health, Englewood, NJ, United States

**Keywords:** case report, chemotherapy, primary SCC, squamous cell carcinoma of the colon, treatment

## Abstract

**Introduction:**

Primary squamous cell carcinoma (SCC) of the colon is an exceptionally rare malignancy (<1% of colorectal cancers). Its infrequency has limited the development of standardized diagnostic and therapeutic guidelines; most evidence derives from individual case reports. Early recognition is challenging because clinical presentation often mimics more common colorectal tumors.

**Case Presentation:**

We describe a 72−year−old woman diagnosed with left−sided (sigmoid) SCC presenting with locally advanced pT4 disease who achieved >12 years of disease−free survival following left hemicolectomy and adjuvant modified FOLFOX−6 chemotherapy. To contextualize this case, we conducted a comprehensive review of 62 published reports meeting strict inclusion criteria. Most patients presented with nonspecific abdominal symptoms. Contrast−enhanced CT(Computed Tomography) and PET/CT (Positron Emission Tomography) were routinely used to define local disease and exclude other squamous primaries. Immunohistochemistry consistently demonstrated positivity for squamous markers (p63, p40, CK5/6) with relative absence of adenocarcinoma-associated markers (CK20, CDX2). Surgery was the primary treatment modality, while systemic chemotherapy was increasingly utilized in stage II–IV disease.

**Conclusion:**

Adjuvant chemotherapy may benefit select high−risk stage II patients with primary colonic SCC with high-risk features such as pT4 tumor penetration. as exemplified in this case. Consistent data collection, collaboration, and molecular characterization are essential to establish evidence−based treatment strategies for this uncommon malignancy.

## Highlights

- Elderly patient with left−sided sigmoid SCC (stage II, T4aN0M0) achieved >12 years disease−free survival after left hemicolectomy and adjuvant mFOLFOX−6.
- Review of 62 reported cases: nonspecific symptoms are common; CT/PET and IHC (p63, p40, CK5/6) are central to diagnosis.
- Surgery remains the cornerstone; systemic chemotherapy is increasingly applied in advanced or high−risk disease.
- Priorities: collaborative registries, molecular profiling, and systematic evaluation of immunotherapy in selected patients.

## Introduction

Squamous cell carcinoma (SCC) of the colon is an exceptionally rare malignancy that accounts for <1% of all colorectal cancers and remains poorly understood due to its low incidence and limited representation in clinical studies (1). In contrast, colon adenocarcinomas arising from glandular cells comprise >90% of colon cancer diagnoses (2, 3). SCCs originate from squamous epithelial cells that are not normally present in the colonic mucosa, posing unique diagnostic and therapeutic challenges (2). The rarity of this entity has impeded the development of standardized pathways for diagnosis, staging, treatment, and surveillance; consequently, most recommendations are extrapolated from colorectal adenocarcinoma and anal canal SCC and supported primarily by case reports and small series (1).

Multiple mechanisms have been proposed to explain the emergence of squamous histology within the colon. A widely cited hypothesis implicates chronic mucosal irritation and inflammation as triggers for squamous metaplasia, with subsequent malignant transformation (4, 5). This is consistent with reports of colonic SCC arising in the setting of ulcerative colitis, Crohn disease, and diverticulitis, where chronic inflammatory stimuli may provide a conducive microenvironment for lineage plasticity (1). Other authors have suggested the presence of multipotent stem cells within colonic crypts that retain the capacity for squamous differentiation, or the migration and expansion of pluripotent basal cells from transitional epithelium near the anorectal junction (5). A viral oncogenic driver such as high−risk human papillomavirus (HPV) well established in anal SCC has been investigated, but most colonic SCC cases, including our index patient, are HPV−negative on p16 immunostaining and/or *in situ* hybridization, making a causal role less likely (4).

From a diagnostic standpoint, distinguishing primary colonic SCC from alternative entities is critical. The classic Williams et al. criteria require exclusion of: (1) squamous−lined fistula tracts, (2) metastasis from a distant squamous primary (e.g., cervix, lung, esophagus, skin), and (3) direct extension from anal squamous epithelium (6). In practical terms, this necessitates a combination of endoscopic assessment, cross−sectional imaging (contrast−enhanced CT), and, when needed, whole−body metabolic staging with PET/CT to exclude extra−colonic primaries (3, 7, 8). Immunohistochemistry (IHC) further supports the diagnosis: p63 and p40 are highly specific for squamous differentiation, often accompanied by CK5/6 positivity; conversely, CK20 and CDX2, which are typical of colorectal adenocarcinoma, are generally absent or only weakly expressed in *pure* squamous tumors. (8, 9−10) This immunophenotypic profile together with negative neuroendocrine markers (chromogranin A, synaptophysin, CD56) helps rule out mimics such as adenosquamous carcinoma and neuroendocrine carcinoma (11).

Therapeutically, surgery remains the cornerstone for localized disease, with hemicolectomy or segmental colectomy favored to achieve negative margins (R0 resection) and adequate nodal harvest (12). Historically, many cases were managed with resection alone; however, contemporary practice is increasingly multimodal, incorporating adjuvant or palliative chemotherapy most commonly FOLFOX and selective radiotherapy for locally advanced or node−positive presentations (13–16). The role of immunotherapy is of growing interest, particularly in reports of PD−1 blockade achieving disease control in microsatellite−stable (MSS/pMMR) tumors when combined with chemotherapy, although data remain preliminary (17, 18).

Despite these evolving strategies, robust evidence to guide stage−specific recommendations is lacking. The heterogeneous and retrospective nature of the literature means that clinicians often individualize care based on tumor biology (grade, serosal invasion), surgical pathology (margin and nodal status), and patient factors, applying principles from high−risk stage II and stage III colon adenocarcinoma where appropriate. Within this context, our report contributes an instructive example: a left−sided (sigmoid) T4aN0M0 primary colonic SCC successfully treated with left hemicolectomy followed by adjuvant mFOLFOX−6 chemotherapy, with > 12 years of disease−free survival (**Table 1**). We pair this case with a narrative review of 62 published reports to contextualize patterns in presentation, diagnostic work−up, immunophenotype, and outcomes, and to highlight pragmatic considerations for adjuvant therapy in high−risk stage II disease.

**Table 1:** Reported cases of primary colonic squamous cell carcinoma (SCC) in the literature, including patient demographics, tumor characteristics, interventions, treatments, and outcomes, alongside the present case.

Paper	Age (years)	Sex	Stage	Right/left side	CEA	Interventions	Treatment	Outcome	
Our Case	82	Female		Left	<5 ng/dL	Surgery	Systemic - FOLFOX	Stable
Singhal et. al., 2021 (19)	65	Female		Right	7.2 ng/mL	Surgery	Systemic - FOLFOX	Alive at 1 month post-op
Garro-Mendiola A et. al., 2023 (4)	59	Female		Left		Surgery	Systemic - CarboTaxol, XRT, 5-FU, cisplatin	Alive
Ramachandra et. al., 2022 (9)		Male		Left	1.7 ng/mL	Surgery	Systemic - CarboTaxol	Alive at 6 months
Jahromi, 2020 (14)	74	Male		Left		Surgery	Systemic - FOLFOX	
Condurache et. al., 2021 (16)	59	Female	T2N0M0 = Stage I	Left		Surgery	XRT, 5-FU, mitomycin C	Alive
Linardoutsos et. al., 2020 (12)	69	Female	pT4pN1 = Stage IIIB	Right		Surgery	Systemic - Unknown Chemotherapy	Developed progressive disease post-op
Liu et. al., 2023 (17)	76	Female	cT4aN2aM1b = Stage IVB	Right		Surgery	Systemic - Immunotherapy, Systemic - FOLFOX	PFS 8.5 months
Haouane et. al., 2023 (2)	40	Male	pT3N0M0 = Stage IIA			Surgery	No therapy	
Sidhu & Grewal, 2024 (20)	54	Male	T2N0M0 = Stage I	Right		Surgery	No therapy	Stable
Abdelqader et. al., 2017 (15)	27	Female		Right		Surgery	No therapy	Alive at 2.5 years
Abdelqader et. al., 2017 (15)	44	Male		Right		Surgery	XRT, Systemic - CarboTaxol, 5-FU	Died 3.5 months after chemoradiation
Husain et. al., 2020 (21)	54	Female		Left		Surgery		Died
Samo et. al., 2015 (22)	81	Female					Cisplatin, 5-FU, XRT		Lost to follow-up
Miyamoto et. al., 2007 (3)	89	Male		Left		Surgery	No therapy	Died of multiple liver metastases and cachexia at 3 months post-op
Recinos et. al., 2020 (10)	61	Female	pT4bN1b = Stage IIIC	Right		Surgery	No therapy	
Albandar & Aljarayhi, 2024 (7)	59	Female		Right		Surgery	Systemic - Unknown Chemotherapy	Disease progression post-op
Cornwell et. al., 2024 (23)	47	Female	pT4bN1b = Stage IIIC	Left	4.6 ug/L	Surgery	XRT	Recurrence in 8 months
Nassar et. al., 2022 (1)	53	Male	pT4aN0M0 = Stage IIB	Left		Surgery	GemCarbo	Died due to disease progression in 10 months
Sahoo et. al., 2022 (24)	56	Male	pT3N0Mx = Stage II	Right	2.41 ng/mL	Surgery	Systemic - Unknown Chemotherapy	Alive and stable
Mondal, 2009 (25)	34	Male		Right		Surgery	Systemic - Unknown Chemotherapy	Stable at 2 years
Bouzroud et al., 2021 (26)	46	Female		Right		Surgery	No therapy	Died from sepsis post surgery
Toumi et.. al., 2018 (13)	46	Female	T3N2aM0 = Stage IIIB	Right		Surgery	Systemic - FOLFOX	Stable at 18 months
Zhao et. al, 2017 (11)	58	Male		Right		Surgery	Gemcitabine, oxaliplatin, capecitabine		PFS 10 months
Wu et. al., 2023 (8)	41	Female	T4	Right		Surgery	capecitabine, oxaliplatin	PFS 7 months
Rajan et. al., 2014 (27)	77	Male	Stage I or II	Right	10 ng/mL	Surgery	No therapy	Died in one month
Jukić et. al., 2011 (28)	59	Female		Right		Surgery	5-FU	Recurrence in 6 months
Hu et. al., 2025 (29)	67	Male		Left		Surgery	ceftriaxone	Died due to multiple organ failure in three months
He et. al., 2021 (18)	59	Male	pT4aN2M0 = Stage IIIC				capecitabine, sintilimab, bevacizumab	Stable
Li et. al., 2022 (30)	56	Female	pT4bN0M0 = Stage IIC	Left		Surgery	Systemic - Unknown Chemotherapy	Died after 14 months of treatment
Hicks & Cowling, 1955 (31)	90	Female		Right		No surgery	No therapy	Died of disease at 1 month
Larizadeh & Powell, 1965 (32)	44	Female		Right		Surgery	No therapy	Alive at 8 months
Minkowitz, 1967 (33)	49	Female		Right		Surgery	No therapy	Died at 5 months
Gaston, 1967 (34)	67	Male		Right		No surgery	No therapy	
Pemberton & Lendrum, 1968 (35)	65	Female		Right		Surgery	No therapy	Alive at 2 years
Birnbaum, 1970 (36)	82	Male		Right		Surgery	No therapy	
Lewis et. al., 1971	61	Male		Right		Surgery	No therapy		Died 10 days post-op
Balfour, 1972 (37)	63	Male		Left		Surgery	No therapy	Alive at 18 months
Horne & McCulloch, 1978 (38)	53	Male		Right		Surgery	No therapy	Died at 11 months
Crissman, 1978 (39)	72	Male				Surgery	No therapy	Died 3 days post-op
Burgess et. al., 1979 (40)	43	Male		Right		Surgery	No therapy	Died at 1 year
Khan et. al., 1979 (41)	64	Male		Right		No surgery	No therapy	
Hickey & Corson, 1981 (42)	48	Female		Left		Surgery	No therapy	Alive at 21 months
Petrelli et. al., 1981 (43)	73	Male		Left		Surgery	No therapy	Died 9 days post-op
Hey & Brandt, 1982 (44)				Right		Surgery	No therapy	Alive at 2 months
Lyttle, 1983 (45)	65	Female		Right		Surgery	No therapy	Alive at 2 months
Gould et. al., 1983 (46)	56	Male		Left		Surgery	No therapy	Died after 3 months
Michelassi, 1988 (47)				Right		Surgery	No therapy	Survival 18 months
Michelassi, 1988 (47)				Right		Surgery	No therapy	Survival 1 month
Balsano, 1985 (48)	65	Male		Right		Surgery	No therapy	
Wyatt et. al., 1991 (49)	71	Male		Right		Surgery	No therapy	Alive at 1 year
Betancourt et. al., 1992 (50)		Female				No surgery	No therapy	Alive 1 year
Vignale et. al., 1993 (51)	69	Male		Left		Surgery	No therapy		Alive at 8 months
Yoshida et. al., 1994 (52)	51	Male		Left		Surgery	No therapy	Died 39 days from diagnosis
Vraux et. al., 1994 (53)				Right		No surgery	Systemic - Unknown Chemotherapy	Died after 5 years
Alekseev et. al., 1994 (54)	69	Male		Left		No surgery	No therapy	5 years' survival
Juturi et. al., 1999 (55)	61	Female		Right		Surgery	Systemic - Unknown Chemotherapy	Alive with no evidence of disease 18 years after diagnosis
Juturi et. al., 1999 (55)	67	Male		Right		Surgery	Systemic - Unknown Chemotherapy	Died of disease at 15 months
Goodfellow et. al., 1999 (56)	66	Male		Right		Surgery	No therapy	
Copur et. al., 2001 (57)	54	Male		Left		Surgery	Systemic - Unknown Chemotherapy	
Fahim et. al., 2006 (58)	68	Female		Left		No surgery	No therapy	Died 3 months after diagnosis
Ngo et. al., 2014 (59)	53	Male		Left		Surgery	Systemic - Unknown Chemotherapy	Alive
Ahmad et. al., 2015 (60)	46	Female		Right		Surgery	No therapy	Alive at 12 month follow up
Mohamed, 2017 (61)	54	Male		Left		Surgery	No therapy	Alive at 12 month follow up

## Methods

### Eligibility criteria

Inclusion. Case reports/series describing primary colonic SCC with adequate diagnostic work−up to exclude metastasis or adenosquamous carcinoma; English full text (or extractable case−level data from included citations); adults (≥ 19 years); reports containing clinical, pathologic, treatment, or outcome data.

Exclusion. Pediatric cases; secondary colonic involvement by non−colonic SCC; squamous differentiation within adenocarcinoma (i.e., not pure SCC); non−English without extractable case data.

### Search strategy

We searched PubMed (last accessed September 30, 2025) using combinations of: “squamous cell carcinoma of the colon,” “primary SCC,” “squamous cell carcinoma,” “treatment,” and “case report.” Filters: English, adults, case reports, free full text. Reference lists of retrieved articles were hand−searched for additional cases. No publication year limits.

### Study selection and data extraction

Two reviewers independently screened titles/abstracts and full texts. Extracted variables included: age/sex; tumor stage; laterality/location; CEA; surgery; systemic therapies and regimens; and outcomes (overall survival, recurrence, progression−free survival when available). When treatment information was not reported, it was assumed absent; when adjuvant therapy was described without explicit surgery, surgery was inferred. These assumptions were applied consistently and acknowledged as limitations.

Data from 33 cases were abstracted from Linardoutsos et al. (2020) and supplemented with individual case details were available. Attempts were made to contact corresponding authors for missing long−term outcomes. (**Table 2**).

**Table 2:** Summary of data extraction method.

Step	Number of reports
Records identified via PubMed database	30
Records excluded after screening	16
Full-text articles assessed	14
Full-text articles excluded	6
Additional records identified via references	48
Total included	62

### Data synthesis

Given rarity and heterogeneity, we performed descriptive synthesis. Treatments were analyzed by stage and modality (surgery, chemotherapy, radiation). Case−level data were tabulated to facilitate pattern recognition.

## Case presentation

A 72−year−old woman with osteoporosis presented in 2012 with intermittent, non−radiating, sharp left lower−quadrant (LLQ) abdominal pain. The discomfort had been evolving over several weeks without associated red−flag symptoms: she denied weight loss, anorexia, fevers, gastrointestinal bleeding, altered bowel habits, nausea, or vomiting. She had no significant family history of cancer and was a non-smoker and consumed no alcohol. Importantly, she had not undergone age−appropriate screening colonoscopy. Physical examination was largely unremarkable apart from mild LLQ tenderness without peritoneal signs. Laboratory testing revealed iron−deficiency anemia (hemoglobin 10 g/dL), prompting endoscopic evaluation; serum CEA was <5 ng/mL.

Colonoscopy demonstrated a 4.8 cm ulcerated, friable mass in the sigmoid colon with an irregular, infiltrative base and surrounding mucosal erythema. There was no diverticulosis, diverticulitis, or endosalpingiosis. Multiple biopsies revealed invasive, poorly differentiated squamous cell carcinoma. The IHC panel (**Table 3**) supported squamous differentiation (p63 positive) with only weak CK20 and very weak CDX2 expression, and was negative for CK7, S100, chromogranin A, synaptophysin, and CD45; CD56 showed only focal staining. High−risk HPV *in-situ* hybridization (ISH) was negative, and p16 was negative, arguing against an HPV−driven neoplasm.

**Table 3:** Immunohistochemistry panel for our patient. .

Marker	Result	Interpretation	Clinical relevance
p63	Positive	Supports squamous differentiation	Helps to distinguish SCC from a poorly differentiated adenocarcinoma
CK20	Weak	Typically positive in colorectal adenocarcinomas	Loss or weak expression of CK20 in CRC is often associated with a higher tumor grade and more aggressive features.
CDX-2	Very weak	Inconclusive, weakly supports colorectal origin	Not expected to be positive in SCC tumors. Loss of CDX-2 expression is a marker of a more aggressive CRC with poor differentiation, advanced stage, and an unfavorable prognosis
p16	Negative	Argues against HPV-driven neoplasia	In certain cancers, such as head and neck squamous cell carcinomas, p16 overexpression is a strong indicator of HPV involvement and is associated with a better prognosis and different treatment protocols.
HPV ISH	Negative	High-risk HPV not detected	This confirms the p16 result and suggests the tumor is likely not HPV-related, which can influence staging and treatment decisions for certain primary sites.
CK7	Negative	Typically absent in colorectal tumors	The CK7-/CK20+ immunoprofile is characteristic of CRC and aids in distinguishing primary lung cancer from CRC metastatic to the lungs.
Chromogranin A/Synaptophysin	Negative	Rules out neuroendocrine tumor	This is important for differential diagnosis as treatment strategies for neuroendocrine tumors are distinct from carcinomas.
CD45	Negative	Rules out lymphoma	This effectively rules out lymphoma as a potential diagnosis, narrowing the possibilities to a carcinoma or another non-hematopoietic malignancy.

Staging studies included contrast−enhanced CT, which identified a mid−to−distal sigmoid mass with possible mesenteric lymphadenopathy but no distant metastases. To exclude an alternative squamous primary, PET/CT was performed and did not reveal extra−colonic disease, thereby satisfying key diagnostic criteria for primary colonic SCC when integrated with histology and clinical evaluation. Routine metabolic and hepatic panels were within normal limits.

Management was discussed at a multidisciplinary cancer conference, and the consensus favored definitive surgery upfront. The patient underwent left hemicolectomy with regional lymphadenectomy. Intraoperatively, the tumor appeared serosally invasive without peritoneal carcinomatosis or liver surface lesions. Gross resection margins were clear. Final pathology confirmed poorly differentiated SCC with invasion through the colonic wall and involvement of the serosal/peritoneal surface (T4a), negative margins, and no lymphovascular or perineural invasion. All examined mesenteric lymph nodes were negative for metastasis (N0), establishing TNM stage T4aN0M0 (Stage II).

Given the tumor’s high−risk feature of serosal invasion (T4a) and the limited evidence base for stage−specific management in colonic SCC, the cancer conference multidisciplinary team recommended adjuvant systemic chemotherapy with modified FOLFOX−6 (mFOLFOX−6). The patient completed 12 cycles without dose reductions. Treatment−related toxicities (e.g., transient neutropenia, sensory neuropathy, mild fatigue) were grade 1–2 and self−limited.

**Figure 1:**
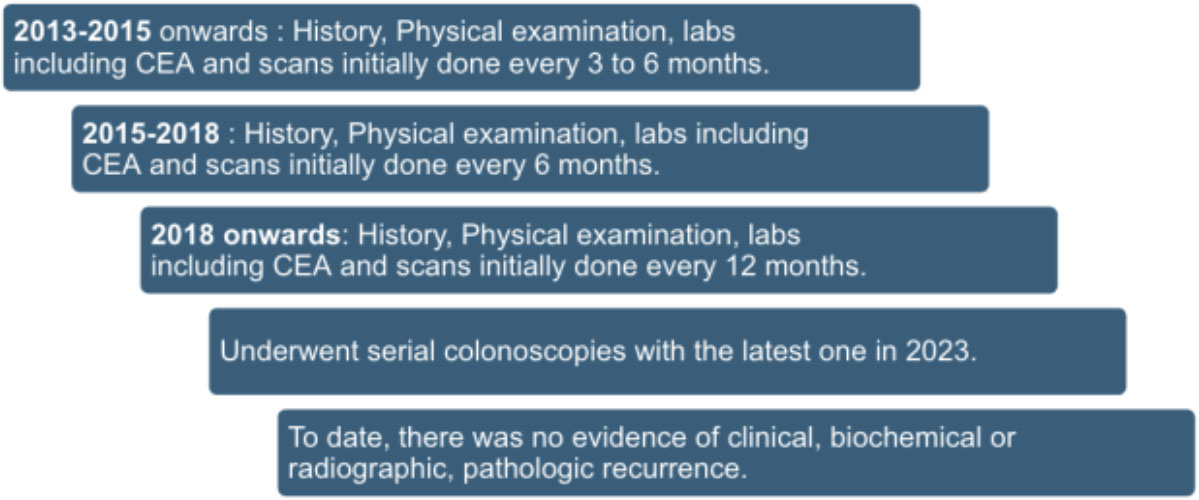
Clinical timeline for our patient.

Post−therapy, the patient entered a structured surveillance program modeled on high−risk colon cancer: history, physical examination, CEA, and cross−sectional imaging every 3–6 months for the first 2 years, every 6 months for years 3–4, and annually thereafter. Colonoscopy at recommended intervals revealed no polyps or recurrent neoplasia, including a 2023 colonoscopy that was negative. To date, she remains disease−free >12 years after definitive treatment with sustained performance status (**Figure 1**).

### Literature review

The literature on primary SCC of the colon remains limited to single−patient case reports and a few small series, reflecting both the rarity of the disease and the lack of uniform reporting guidelines. Our review adds to this field by synthesizing 62 published cases from 1955 to September 2025. This dataset, spanning 70 years, has a broad temporal window that captures significant evolution in diagnostic capability and treatment approaches. In earlier decades, imaging was less advanced (e.g., PET/CT scans became available in 2010) and long−term follow−up was infrequent; more recent reporting provides greater detail on surgical pathology, immunohistochemistry, and systemic therapies.

### Clinical presentation trends

Across cases, the majority of patients presented with abdominal pain (≈70%), followed by altered bowel habits, weight loss, or anemia. A particularly notable and recurring pattern is the frequency of obstructive symptoms, with several cases requiring urgent decompression or emergent surgery. Fewer than 10% of cases were diagnosed during routine screening colonoscopy, emphasizing the need for vigilance when encountering unusual or non−adenomatous features endoscopically.

Age at diagnosis is typically clustered between 50–75 years, although younger presentations have been described, including individuals in their third and fourth decades, often in association with underlying inflammatory bowel disease. Older females were slightly more represented in left−sided tumors, similar to our index patient.

### Tumor distribution and staging

Historically, the right colon (cecum and ascending colon) appeared overrepresented in case reports, though more recent literature reveals a more even distribution with increasing recognition of sigmoid and left−sided tumors mirroring advancements in colonoscopy and diagnostic accuracy (3, 9). Most patients were diagnosed at stage II or III, with nodal metastases documented in 30–50% of cases and distant metastases most commonly to liver, lung, or peritoneum in 10–20%.

Poor differentiation was predominant across reports, reinforcing the aggressive phenotype of SCC in the colon. Perforation and abscess formation were recurrent complications, raising the possibility that the inflammatory tumor microenvironment may contribute to the pathologic process.

### Diagnostic evolution

Use of PET/CT has significantly increased since 2010, improving clinicians’ ability to confirm colonic primary origin by excluding more common SCC primaries (gynecologic, pulmonary, cutaneous). Likewise, widespread adoption of IHC including p40/p63 has strengthened diagnostic confidence, reducing historical uncertainty regarding whether early cases truly represented SCC rather than poorly differentiated adenocarcinoma.

### Long−term outcomes

Survival outcomes are inconsistently reported across the literature, with historical cases demonstrating high mortality, often within 1–3 years of diagnosis. More recent reports, reflecting advances in multimodality therapy, describe long-term survival exceeding 10 years, as observed in our patient. Interpretation of these trends is limited by short follow-up durations and incomplete outcome reporting, particularly in earlier case reports. When survival duration or recurrence data were not explicitly reported, outcomes were recorded as unavailable rather than inferred, and these cases were included in the descriptive synthesis but excluded from longitudinal interpretation. Although attempts were made to contact corresponding authors for missing long-term outcomes, substantial gaps remained. Consequently, reported long-term survival reflects a subset of cases with extended follow-up, introducing survivorship bias and requiring cautious interpretation of apparent improvements over time.

Collectively, these findings highlight the need for standardized documentation and follow−up for future cases. Prospective registry−based reporting would substantially enhance understanding of this entity.

### Imaging

Radiologic imaging is pivotal not only for identifying colonic SCC but also for differentiating it from more common colorectal malignancies and for staging to exclude alternative primaries. In our review, contrast−enhanced CT was used in nearly all reported cases and typically demonstrated an infiltrative, sometimes necrotic mass involving the bowel wall, with potential extension into adjacent fat planes or peritoneum (21, 23). Obstructing lesions were not uncommon, with a subset presenting emergently due to large bowel obstruction (9). CT was also crucial for detecting complications such as contained perforation, abscess, or fistulization, which have been documented more frequently in SCC than adenocarcinoma in some reports.

Colonoscopy remains the first−line diagnostic modality, often revealing a fungating or ulceroproliferative mass that may hinder complete luminal assessment (9). In cases with obstructive tumors, stent placement or diverting procedures have been performed prior to definitive surgical management (15).

PET/CT serves an essential role in excluding alternative SCC primaries, given the colon is an atypical site for primary squamous disease. Its use has expanded in recent literature, particularly as a confirmatory tool when biopsy shows squamous morphology. PET/CT can detect clinically occult metastases in up to one−third of advanced cases and may influence operability decisions (7, 8).

MRI has been selectively used in tumors with suspected pelvic involvement or to assess depth of invasion when CT is equivocal (4). This may be particularly relevant in rectosigmoid tumors, where precise staging influences whether neoadjuvant therapy should be considered a strategy occasionally extrapolated from anal SCC protocols.

Overall, multimodal imaging facilitates accurate diagnosis, appropriate staging, and surveillance of primary colonic SCC, contributing to postoperative monitoring for recurrence especially during the first five years when relapse risk is highest.

### Tumor markers and immunophenotype

Serum tumor markers such as CEA and CA 19−9 have shown inconsistent results in colonic SCC. Although markedly elevated CEA has been reported in isolated cases (4), many remain within normal limits (24), limiting its sensitivity and utility as a standalone marker (9). As such, routine markers commonly used for colorectal adenocarcinoma should not be relied upon to triage suspected SCC.

In contrast, IHC is indispensable. Nuclear p63 and p40 positivity was observed in the majority of published cases (4, 8, 10, 11, 26) is highly supportive of squamous differentiation. CK5/6 positivity further strengthens this characterization (10, 11). Frequently, tumors demonstrate high proliferation indices (e.g., Ki−67 >70%), correlating with aggressive histologic features (4, 8, 11, 26).

Markers typical of intestinal lineage (CK20, CDX2) are typically negative or only focally expressed in pure SCC, allowing distinction from adenocarcinoma with squamous differentiation (9, 11). Neuroendocrine markers (chromogranin A, synaptophysin, CD56) are likewise negative, preventing misclassification as high−grade neuroendocrine carcinoma (26).

Select reports have described PD−L1 expression, with implications for experimental use of checkpoint inhibitors in advanced or refractory disease (8). The majority of tumors tested remain microsatellite stable (MSS/pMMR) (4, 8), consistent with the poor correlation between high−level genomic instability and squamous differentiation in this context.

Finally, while HPV−driven oncogenesis is a hallmark of anal SCC, HPV testing has been negative in most primary colonic SCC cases, including our own (4), arguing against viral contribution in the colon. The occasional p16 expression in some cases likely reflects cell−cycle dysregulation rather than viral etiology (11).

### Histopathology

Histological diagnosis of primary SCC of the colon relies on recognition of features characteristic of squamous differentiation. These include: Keratinization with keratin pearls, intercellular bridges between tumor cells, eosinophilic cytoplasm, marked nuclear atypia and mitotic activity (25, 51).

Most reported cases, including ours, describe moderately to poorly differentiated tumors (4, 11, 21), consistent with historically adverse prognosis. Areas of necrosis and infiltrative growth patterns are also frequently noted, reflecting the tumor’s aggressive nature.

A major pathological distinction must be made between primary pure SCC and adenosquamous carcinoma, the latter demonstrating both glandular and squamous components. This is crucial because treatment principles and biological expectations differ. In our case, the absence of glandular morphology and negative CK20/CDX2 staining confirmed a pure SCC phenotype.

To ensure accurate classification, pathologists follow Williams et al. diagnostic criteria (6), which exclude: 1. Squamous−lined fistula tracts 2. Metastatic SCC from an extra−colonic primary 3. Direct extension from anal canal SCC.

Contemporary reports emphasize that this diagnostic rigor be supported by comprehensive imaging (CT/PET) and complete clinical history. Additionally, tumor staging particularly identification of T4 disease and nodal involvement, is paramount for prognostication and selection of adjuvant therapy (30).

The constellation of findings in our patient- poor differentiation, T4a invasion, and negative lymph nodes informed subsequent management decisions and correlated with her favorable long−term outcome.

### Analysis

This case highlights several diagnostic and therapeutic themes that recur across the published literature on primary colonic SCC. First, the clinical presentation of nonspecific abdominal pain and iron−deficiency anemia in the absence of changes in bowel habits or overt gastrointestinal bleeding mirrors many reported cases and underscores why diagnosis is often delayed until colonoscopy and tissue sampling are performed (1, 3). In our patient, the lack of prior screening colonoscopy likely contributed to presentation at a locally advanced T4a stage, a scenario commonly observed in narrative reviews and single−institution experiences (12).

Second, the pathological and immunophenotypic profile is representative of *pure* squamous histology. The strong p63 positivity, coupled with negative CK7 and neuroendocrine markers and only weak CK20/CDX2, supports squamous differentiation and effectively argues against colorectal adenocarcinoma, adenosquamous carcinoma, and neuroendocrine carcinoma (8–10). Anal cell cancer tends to be HPV positive as noted by p16 or ISH positivity. In contrast, our case demonstrated HPV negativity (p16-/ISH-), a finding reported in the majority of published primary colonic SCC cases (4 of 5 references). This consistent HPV-negative profile reinforces the emerging consensus that primary colonic SCC is biologically distinct from HPV-driven anal SCC (p16/ISH+), despite overlapping squamous markers (8, 11).

Third, with respect to staging and exclusion of mimics, our diagnostic pathway adhered to the Williams criteria through systematic anatomic and metabolic imaging to rule out alternate primaries and direct extension (6). The incorporation of PET/CT used in several contemporary reports adds confidence in establishing colonic origin by surveying typical squamous primary sites (gynecologic, pulmonary, cutaneous) and detecting otherwise occult disease (3, 7, 8).

From a therapeutic standpoint, this case informs the practical question of adjuvant chemotherapy in stage II colonic SCC. In colorectal adenocarcinoma, T4 invasion is a recognized high−risk feature for which adjuvant therapy is often recommended. While colonic SCC lacks randomized data, the pattern of care in modern reports suggests increasing use of FOLFOX−based regimens in stage II disease with high−risk features and in nearly all stage III cases (12–14). Our patient’s durable >12−year disease−free survival following mFOLFOX−6 adds to this signal and supports a stage−adapted, risk−stratified approach in which T4a pathology justifies postoperative chemotherapy even in the absence of nodal involvement.

The role of radiotherapy remains selective. While some node−positive or locally advanced presentations have received chemoradiation (e.g., with 5−FU/mitomycin C or platinum−taxane backbones) (16, 62), routine use for completely resected, node−negative colon primaries is not supported by current case−based evidence and is generally reserved for margin−threatened disease or pelvic fixation. In our patient, negative margins and absence of nodal or perineural/lymphovascular invasion argued against adjuvant radiation.

Emerging data regarding immunotherapy in MSS/pMMR colonic SCC are intriguing but preliminary. Case reports describe partial responses to PD−1 blockade combined with chemotherapy in metastatic or unresectable settings (17, 18), suggesting that PD−L1 expression and other tumor−immune features may have future relevance in selected patients (8). At present, however, these observations should be viewed as hypothesis−generating and not as standards of care.

Finally, this case emphasizes the importance of structured, long−term surveillance. Many historical reports suffer from abbreviated follow−up; recurrences, when documented, tend to occur within the first few years. Our patient’s >12−year remission demonstrates the curative potential of margin−negative resection plus appropriately selected adjuvant therapy in high−risk stage II disease. The trajectory of surveillance we employed—more intensive during the first 3–5 years, followed by annual assessments—mirrors patterns used in colon adenocarcinoma and appears reasonable for colonic SCC in the absence of disease−specific guidelines.

In summary, this case reinforces several pragmatic points for clinicians: (1) maintain diagnostic vigilance for SCC when endoscopic and histologic findings are discordant with adenocarcinoma; (2) apply a multimodal diagnostic work−up to establish primary colonic origin; (3) consider adjuvant FOLFOX for stage II T4a tumors after R0 resection; and (4) adopt a prolonged surveillance schedule given uncertainties about late recurrence. These principles, while derived from limited evidence, are consistent with patterns across 62 published reports and provide a rational framework for decision−making until higher−level data become available.

### Treatment

#### Surgical intervention

Surgical resection remains the definitive modality for localized disease, with hemicolectomy or segmental colectomy used to achieve negative margins and obtain adequate nodal sampling. In our analysis, >90% of patients underwent surgery, particularly in studies published since 2000. Historical exceptions (non−operative management or palliative measures only) typically correlated with advanced disease, metastasis, or presentation prior to modern staging standards (31, 34, 41, 50, 58).

While colectomy is standard, technique can vary according to tumor location: right hemicolectomy for cecal or ascending tumors, left hemicolectomy for descending or sigmoid tumors, and anterior resection for rectosigmoid lesions.

The core principle of curative surgery for advanced or recurrent pelvic malignancies, including SCC, is achieving an R0 resection. An R1 (positive margin) resection is a major prognostic factor for poor outcomes, including increased local recurrence and decreased survival. Pelvic fixation techniques, such as those involving sacrectomy or bony pelvic sidewall resection, are a component of extended pelvic exenteration (a radical, multi-organ removal surgery) used to physically remove tumor that is fixed to or invading bony structures, thus maximizing the chance of a complete tumor clearance. Given the significant morbidity, mortality, and complex recovery associated with these extended procedures, the indications are very selective and decided on a case-by-case basis by a multidisciplinary team at high-volume centers.

Specific indications for considering an extended resection with pelvic fixation after an R1 margin are limited to scenarios where:

- Microscopic positive margins involve bony structures: The R1 margin is confirmed to be at the interface of the resected specimen and the pelvic bone (sacrum, ilium, or ischium), and further, more extensive bony resection (sacrectomy or lateral wall resection) could potentially clear this margin.
- Absence of extrapelvic metastatic disease: Radical surgery is only considered when the disease is confined to the pelvis. The presence of distant metastases is generally a contraindication for curative-intent radical surgery, shifting the focus to palliative care.
- Favorable patient condition: The patient must be fit enough to withstand the physiological stress of a major operation and its prolonged recovery period.
- Potential for R0 achievement: There must be a high likelihood that the extended resection will convert the R1 status to an R0 status.
- Managing intractable symptoms: In some cases, even if R0 is unlikely, a limited bony resection might be considered to palliate intractable pain, bleeding, or obstruction caused by the fixed tumor, but this is a different indication than curative-intent surgery.

Rare instances of multivisceral resection have been required when direct invasion into adjacent structures, such as bladder or abdominal wall was identified (21, 23).

#### Adjuvant chemotherapy

A major evolving theme in the literature is increasing use of adjuvant chemotherapy, even in stage II disease when high−risk features exist. High-risk stage II colon cancer signifies the tumor has grown deeper (pT4-Tumor penetrating the bowel wall) or has concerning features like perforation, obstruction, poor differentiation, or perineural or lymphovascular invasion, or fewer than 12 lymph nodes were examined. These factors increase recurrence risk, influencing decisions for adjuvant chemotherapy, and the duration of adjuvant therapy course. FOLFOX emerged as the most frequently used modern regimen, likely due to its success in node−positive and high−risk stage II adenocarcinoma and its tolerability in older adults and showing superiority to 5FU/leuvocorin (13, 14). The addition of oxaliplatin provided longer disease free interval and lower risk of cancer recurrence. Several reports describe prolonged disease−free survival including our patient and others treated with postoperative FOLFOX or XELOX.

Alternative regimens include carboplatin−paclitaxel, occasionally selected for tumors with high keratinizing phenotype or concern for shared biology with anal SCC (4, 9). Agents such as cisplatin + 5−FU have also been used, particularly when combined with radiation therapy (22).

The available data increasingly support risk−adapted use of chemotherapy in Stage II, particularly when T4 invasion, perforation, or poor differentiation are present. Nodal involvement (Stage III) nearly always prompted chemotherapy in more recent cases (12).

#### Radiation therapy

Radiation therapy (XRT) remains selectively used. Its role appears most appropriate in:node−positive presentations, locally advanced pelvic involvement, margin−positive resections, basaloid or anal canal−adjacent histology.

Combination chemoradiation has shown benefit in downstaging locally advanced tumors and improving symptom relief in unresectable cases, though robust long−term data are lacking (16, 22).

Routine use of adjuvant radiation in fully resected, node−negative colon SCC is not supported by current case−based evidence.

#### Systemic therapy for metastatic disease

Outcomes in metastatic SCC remain poor, though recent success with checkpoint inhibitors in MMS tumors is noteworthy. Two modern case reports documented partial responses to PD−1 blockade combined with platinum−based chemotherapy, suggesting potential synergy (17, 18). Ongoing advances in molecular phenotyping including PD−L1 expression and tumor mutational profiling may identify biomarkers that predict immunotherapy responsiveness.

#### Treatment patterns over time

Over the past several decades, there has been a clear evolution in the management and reporting of primary colonic SCC. Before the year 2000, most published cases reflected a limited diagnostic workup often relying on basic imaging and histology without routine use of immunohistochemistry or metastatic exclusion studies such as PET/CT. (31, 34, 41, 50, 58). Treatment during this era was predominantly surgery alone, and adjuvant therapy was rarely considered due to the absence of evidence-based guidance and limited staging tools (43, 47, 48). Reporting of outcomes was also inconsistent, with many patients experiencing early recurrence and high mortality within the first few years following diagnosis (37). In contrast, after 2000, widespread adoption of modern oncologic principles has led to significant advances in diagnostic accuracy and therapeutic strategy. Recent literature increasingly supports multimodal treatment involving surgical resection combined with systemic chemotherapy, such as FOLFOX or platinum-based regimens and selective use of radiation therapy for locally advanced disease (13–16, 22). Improved diagnostic specificity through routine use of IHC markers (p63, p40, CK5/6) and PET/CT for exclusion of alternative primaries has strengthened differentiation of true colonic SCC from mimics (3, 7, 8). Additionally, modern reports more consistently document durable remissions, with some patients achieving >10-year survival following aggressive multimodal therapy. (13, 23, 30) These trends collectively suggest that utilizing contemporary colorectal cancer treatment paradigms may meaningfully improve survival in this historically aggressive and understudied malignancy.

## Conclusion

Primary colonic SCC, though rare, should be considered when pathology is inconsistent with adenocarcinoma. Surgical resection remains foundational; adjuvant therapy should be individualized, with chemotherapy favored for high−risk stage II and for stage III–IV disease. Multimodal strategies may improve outcomes in selected patients, balanced against quality−of−life considerations.

### Future perspective

Progress hinges on collaborative registries and pooled data. Molecular profiling and next−generation sequencing could reveal targetable pathways or viral associations. Immunotherapy particularly checkpoint blockade in PD−L1−positive or MSI−high tumors warrants systematic evaluation. AI−assisted pathology and radiomics may refine diagnosis, staging, and risk stratification, moving care toward precision oncology.
